# The past, present and future of light-gated ion channels and optogenetics

**DOI:** 10.7554/eLife.42367

**Published:** 2018-10-22

**Authors:** Sheena A Josselyn

**Affiliations:** 1 Program in Neurosciences & Mental HealthHospital for Sick ChildrenTorontoCanada; 2 Department of PsychologyUniversity of TorontoTorontoCanada; 3 Department of PhysiologyUniversity of TorontoTorontoCanada; 4 Institute of Medical SciencesUniversity of TorontoTorontoCanada; 5 Brain, Mind & Consciousness ProgramCanadian Institute for Advanced ResearchTorontoCanada

**Keywords:** optogenetics, light-gated ion channels, channelrhodospins, neural circuits, animal behavior, neuropsychiatric disease

## Abstract

The discovery of the mechanisms underlying light-gated ion channels called channelrhodospins and the subsequent development of optogenetics illustrates how breakthroughs in science and technology can span multiple levels of scientific inquiry. Our knowledge of how channelrhodopsins work emerged from research at the microscopic level that investigated the structure and function of algal proteins. Optogenetics, on the other hand, exploits the power of channelrhodospins and similar proteins to investigate phenomena at the supra-macroscopic level, notably the neural circuits involved in animal behavior that may be relevant for understanding neuropsychiatric disease. This article is being published to celebrate Peter Hegemann, Karl Deisseroth and Ed Boyden receiving a 2018 Canada Gairdner International Award "for the discovery of light-gated ion channel mechanisms, and for the discovery of optogenetics, a technology that has revolutionized neuroscience".

## The award, part I: Light-gated ion channels

Microbial opsin genes encode light-sensitive proteins that are found mostly in archaea and bacteria. Examples include bacteriorhodopsins (which are proton pumps), halorhodopsins (ion pumps) and channelrhodopsins (ion channels). Channelrhodospins are unique in that they are the only class of light-activated ion channels identified in biology to date. They are also the foundations on which the research that is being recognized by one of the 2018 Canada Gairdner International Awards was built.

Over many years, Peter Hegemann and colleagues studied the behavior of the single-celled green alga *Chlamydomonas*. These algae alter the direction in which they swim in response to flashes of light. Based on the fast time course of this phototactic response, Hegemann hypothesized the presence of a single protein with two key components (one to 'sense' the light, and one to 'respond' to the light by initiating an electrical signal that leads to a change in the direction they are swimming). This work culminated in the molecular and functional identification of the first channelrhodopsin gene, encoding a cation channel, by Hegemann (then at the University of Regensburg) and co-workers in 2002 (Nagel et al., 2002). Although the backbone of the channelrhodopsin protein is a familiar 7-transmembrane structure common to many proteins (including the G-protein coupled receptor family of proteins that is targeted by many drugs), channelrhodospins are distinct in their ability to conduct particular ions in response to certain colors of light, all within milliseconds. Precisely how channelrhodopsin accomplished this remarkable feat became a biological mystery.

Over the next fifteen years or so, Hegemann (who moved to Humboldt University in Berlin in 2004), Karl Deisseroth (Stanford University) and colleagues uncovered the functional operating principles of channelrhodopsin, down to the level of determining the individual amino acids that regulate ion flow, gating kinetics and color selectivity (Deisseroth and Hegemann, 2017). The high-resolution crystal structures of the cation-conducting channelrhodopsins were solved earlier this decade (Kato et al., 2012), and the structures of anion-conducting channelrhodopsins have just been solved (Kim et al., 2018; Kato et al., 2018).

These structural insights also enabled a series of further advances: examples include engineered channelrhodopsins that respond to light faster than wildtype channelrhodopsins (Gunaydin et al., 2010); channelrhodopsins that act as bi-stable switches (Berndt et al., 2009); channelrhodopsins that are activated by different wavelengths of light (Zhang et al., 2008; Yizhar et al., 2011); and channelrhodopsin-based chloride channels (Berndt et al., 2014; Wietek et al., 2014). This series of fundamental scientific discoveries examining how channelrhodopsins (and related proteins) respond to light with such speed and precision also led to a new experimental tool, optogenetics, that could be used to control the activity of selected cells deep within systems as complex as the mammalian brain with extraordinary temporal and spatial precision. As the prize citation notes: "optogenetics is a technology that has revolutionized the field of neuroscience".

## The award, part II: Optogenetics

Understanding the brain has been described as one of the final frontiers of science. Of course, there was significant progress towards this goal before optogenetics entered the scientific lexicon. But a step change in the type of scientific questions that could be asked, and the practical ways in which many scientists conduct research, can be traced back to a single paper from Deisseroth's lab in which he and co-workers – Ed Boyden, Feng Zhang (who shared the 2016 Canada Gairdner International Award for his work on CRISPR-Cas and genome editing), along with Ernst Bamberg and Georg Nagel – reported that the activity of neurons in a dish expressing channelrhodopsin could be driven by simply shining a light (Boyden et al., 2005).

Subsequent papers described the development of other, equally important, optogenetic components – tools for targeting opsins (Tsai et al., 2009) and light (Adamantidis et al., 2007) to circuit elements of interest in behaving animals. The development of opsins for neuronal silencing rounded out the optogenetics toolbox (Han and Boyden, 2007; Zhang et al., 2007; Chow et al., 2010; Gradinaru et al., 2010). Optogenetics allows scientists to achieve precise temporal control over the activity of defined populations of cells in the brain of a behaving animal, and thus probe the circuits mediating both brain function and dysfunction.

The development of optogenetics cleanly splits neuroscience, and many other branches of science, into pre- and post-optogenetic eras.

The development of optogenetics cleanly splits neuroscience, and many other branches of science, into pre- and post-optogenetic eras. As with any new technique, the first findings using optogenetics were simply confirmatory. But, as scientists grew past the 'gee whiz' factor and became more adept at designing experiments that fully exploited this new way of investigating brain function, novel – and perhaps previously unknowable – insights began to emerge.

Among the many notable scientific insights enabled by optogenetics, I would include:

- Uncovering how complex behavioral states (such as anxiety) arise from individual state features (including changes in respiration and risk-avoidance behavior), and the brain circuit components underlying these states (Kim et al., 2013). Optogenetics, for the first time, enabled the independent manipulation of the activity of different output projections originating from the same brain region, thereby allowing complex behavioral states to be dissected into component features and mapped across the brain.
- Identifying the long-sought cell ensembles (engrams) underlying memory (Liu et al., 2012), and taking advantage of optogenetic actuators to both increase and decrease activity in the same neurons to discover how these ensembles control the separation or integration of individual memories (Rashid et al., 2016).
- Illuminating the cell ensembles that support basic survival drives and control the most fundamental functions (for example, hunger, thirst, energy balance, respiration, arousal, sleep, circadian rhythm). These circuits contained cell types with diverse (even opposite) functional roles and therefore required the power of optogenetics to resolve them (beginning with Adamantidis et al., 2007).
- Determining specific cells and neural pathways controlling normal and abnormal motor action patterns. A long-standing question in movement initiation was answered with optogenetic experiments which revealed the differential roles of intermixed striatal direct vs. indirect pathways (Kravitz et al., 2010). In addition to increasing our understanding of adaptive movement regulation, this insight also has clinical relevance for Parkinsonism and related disorders.
- Discovering the essential circuit basis of reward; with optogenetics, bursts of activity in midbrain dopamine neurons were finally and formally shown to constitute a reward signal, beginning with relatively simple Pavlovian conditioning (Tsai et al., 2009), and followed by more complex learning tasks (Saunders et al., 2018).
- Revealing the role of the theta rhythm in the hippocampus for both memory encoding and retrieval (Siegle and Wilson, 2014). Although hippocampal theta rhythms had been studied for many years, understanding their contribution to memory processes required both the speed and precision of closed-loop optogenetics so that neuronal activity could be manipulated in different phases of the theta cycle.
- Resolving the precise neural circuit and activity patterns that drive social behavior, including mating and aggression (Lin et al., 2011). This discovery was made possible as optogenetics allowed the manipulation of a very small region deep in the brain (and not adjacent brain regions or fibres-of-passage).

## The future of optogenetics

So what does the future hold? Scientific progress across many fields may be facilitated by the continued development of: i) more efficient opsins that can be customized for any experimental requirement; ii) improved methods for targeting one or more opsins to specific cells; iii) better ways of focusing light onto a single cell or multiple cells in a specific temporal pattern, all in freely-behaving animals. Beyond generating bigger, faster and stronger opsins, optogenetics could also be more fully integrated with other technologies that are used to study circuits in the brain, including electrophysiology and calcium or voltage imaging. There has been notable progress towards this objective. However, to fully appreciate brain function, unraveling the complex circuitry involved is likely to be a necessary, but not sufficient, condition. It may also be crucial to understand the roles of, and the interplay between, the many neurotransmitters, neuromodulators, intracellular signaling and other brain molecules.

Optogenetics ushered in an era of neuroscience in which neuronal circuits became the new basic unit of inquiry. In many ways, the 'optogenetic revolution' echoed, and perhaps to some extent eclipsed, an earlier revolution in molecular neuroscience. Further evolution of neuroscience as field, though, may demand the seamless integration of these two pillars. The development of artificial light-inducible molecules is one way to increase interactions between the circuit and molecular domains (Shemesh et al., 2017). For instance, because of the structural similarities between opsins and G-protein coupled receptor (GPCRs), researchers have been able to replace the intracellular loops of some vertebrate rhodopsins with intracellular loops from specific GCPRs, such that light can then be used to activate certain intracellular pathways (Airan et al., 2009). Moreover, photosensitive plant proteins (that change their conformation in response to light) are now being used to control the actions of many molecules, including those important for CRISPR manipulations (Rost et al., 2017). A molecular read-out of neuronal activity (phosphorylation of the transcription factor CREB) was used as the first proof-of-principle for optogenetics (Deisseroth, 2015), so the time may now be ripe to close the optogenetic/molecular circle of science.

The basic scientific insights into brain function afforded by optogenetics (especially when it is used in combination with other techniques) are almost limitless. Researchers are using a range of tools across many species to interrogate the fundamental underpinnings of both brain function (e.g., from memory to homeostasis) and dysfunction (e.g., from autism to Alzheimer’s disease). The field is also changing rapidly, and one day optogenetics may shed light on the 'hard problems' of neuroscience, such as the neural basis of consciousness. But will optogenetics ever be used to treat disease? Several clinical trials are already underway, the results of which are anticipated eagerly. However, rather than optogenetics being used to treat patients directly, it is more likely that new treatments for brain disorders (and other disorders) will derive from the kinds of fundamental discovery research made possible by optogenetics.

Of course, the fundamental knowledge and potential clinical applications gained by optogenetic experiments would not have been possible without the basic science discoveries on how green algae respond to light and how light-activated proteins work, turning molecular structure into function. The optogenetic revolution clearly illustrates that one never knows from where the next big discovery will come and underlines the critical importance of fundamental curiosity-driven discovery research. I am reminded of the song 'Started from the Bottom' by Drake. One could say that optogenetics started from the bottom (literally, from pond scum); now it is being used in laboratories around the world to shed new light on how that most complex of systems, the brain, works.

## References

[bib1] Adamantidis AR, Zhang F, Aravanis AM, Deisseroth K, de Lecea L (2007). Neural substrates of awakening probed with optogenetic control of
hypocretin neurons. Nature.

[bib2] Airan RD, Thompson KR, Fenno LE, Bernstein H, Deisseroth K (2009). Temporally precise in vivo control of intracellular
signalling. Nature.

[bib3] Berndt A, Yizhar O, Gunaydin LA, Hegemann P, Deisseroth K (2009). Bi-stable neural state switches. Nature Neuroscience.

[bib4] Berndt A, Lee SY, Ramakrishnan C, Deisseroth K (2014). Structure-guided transformation of channelrhodopsin into a
light-activated chloride channel. Science.

[bib5] Boyden ES, Zhang F, Bamberg E, Nagel G, Deisseroth K (2005). Millisecond-timescale, genetically targeted optical control of neural
activity. Nature Neuroscience.

[bib6] Chow BY, Han X, Dobry AS, Qian X, Chuong AS, Li M, Henninger MA, Belfort GM, Lin Y, Monahan PE, Boyden ES (2010). High-performance genetically targetable optical neural silencing by
light-driven proton pumps. Nature.

[bib7] Deisseroth K (2015). Optogenetics: 10 years of microbial opsins in
neuroscience. Nature Neuroscience.

[bib8] Deisseroth K, Hegemann P (2017). The form and function of channelrhodopsin. Science.

[bib9] Gradinaru V, Zhang F, Ramakrishnan C, Mattis J, Prakash R, Diester I, Goshen I, Thompson KR, Deisseroth K (2010). Molecular and cellular approaches for diversifying and extending
optogenetics. Cell.

[bib10] Gunaydin LA, Yizhar O, Berndt A, Sohal VS, Deisseroth K, Hegemann P (2010). Ultrafast optogenetic control. Nature Neuroscience.

[bib11] Han X, Boyden ES (2007). Multiple-color optical activation, silencing, and desynchronization of
neural activity, with single-spike temporal resolution. PLoS One.

[bib12] Kato HE, Zhang F, Yizhar O, Ramakrishnan C, Nishizawa T, Hirata K, Ito J, Aita Y, Tsukazaki T, Hayashi S, Hegemann P, Maturana AD, Ishitani R, Deisseroth K, Nureki O (2012). Crystal structure of the channelrhodopsin light-gated cation
channel. Nature.

[bib13] Kato HE, Kim YS, Paggi JM, Evans KE, Allen WE, Richardson C, Inoue K, Ito S, Ramakrishnan C, Fenno LE, Yamashita K, Hilger D, Lee SY, Berndt A, Shen K, Kandori H, Dror RO, Kobilka BK, Deisseroth K (2018). Structural mechanisms of selectivity and gating in anion
channelrhodopsins. Nature.

[bib14] Kim SY, Adhikari A, Lee SY, Marshel JH, Kim CK, Mallory CS, Lo M, Pak S, Mattis J, Lim BK, Malenka RC, Warden MR, Neve R, Tye KM, Deisseroth K (2013). Diverging neural pathways assemble a behavioural state from separable
features in anxiety. Nature.

[bib15] Kim YS, Kato HE, Yamashita K, Ito S, Inoue K, Ramakrishnan C, Fenno LE, Evans KE, Paggi JM, Dror RO, Kandori H, Kobilka BK, Deisseroth K (2018). Crystal structure of the natural anion-conducting channelrhodopsin
GtACR1. Nature.

[bib16] Kravitz AV, Freeze BS, Parker PR, Kay K, Thwin MT, Deisseroth K, Kreitzer AC (2010). Regulation of parkinsonian motor behaviours by optogenetic control of
basal ganglia circuitry. Nature.

[bib17] Lin D, Boyle MP, Dollar P, Lee H, Lein ES, Perona P, Anderson DJ (2011). Functional identification of an aggression locus in the mouse
hypothalamus. Nature.

[bib18] Liu X, Ramirez S, Pang PT, Puryear CB, Govindarajan A, Deisseroth K, Tonegawa S (2012). Optogenetic stimulation of a hippocampal engram activates fear memory
recall. Nature.

[bib19] Nagel G, Ollig D, Fuhrmann M, Kateriya S, Musti AM, Bamberg E, Hegemann P (2002). Channelrhodopsin-1: a light-gated proton channel in green
algae. Science.

[bib20] Rashid AJ, Yan C, Mercaldo V, Hsiang HL, Park S, Cole CJ, De Cristofaro A, Yu J, Ramakrishnan C, Lee SY, Deisseroth K, Frankland PW, Josselyn SA (2016). Competition between engrams influences fear memory formation and
recall. Science.

[bib21] Rost BR, Schneider-Warme F, Schmitz D, Hegemann P (2017). Optogenetic tools for subcellular applications in
neuroscience. Neuron.

[bib22] Saunders BT, Richard JM, Margolis EB, Janak PH (2018). Dopamine neurons create Pavlovian conditioned stimuli with
circuit-defined motivational properties. Nature Neuroscience.

[bib23] Shemesh OA, Tanese D, Zampini V, Linghu C, Piatkevich K, Ronzitti E, Papagiakoumou E, Boyden ES, Emiliani V (2017). Temporally precise single-cell-resolution optogenetics. Nature Neuroscience.

[bib24] Siegle JH, Wilson MA (2014). Enhancement of encoding and retrieval functions through theta
phase-specific manipulation of hippocampus. eLife.

[bib25] Tsai HC, Zhang F, Adamantidis A, Stuber GD, Bonci A, de Lecea L, Deisseroth K (2009). Phasic firing in dopaminergic neurons is sufficient for behavioral
conditioning. Science.

[bib26] Wietek J, Wiegert JS, Adeishvili N, Schneider F, Watanabe H, Tsunoda SP, Vogt A, Elstner M, Oertner TG, Hegemann P (2014). Conversion of channelrhodopsin into a light-gated chloride
channel. Science.

[bib27] Yizhar O, Fenno LE, Prigge M, Schneider F, Davidson TJ, O'Shea DJ, Sohal VS, Goshen I, Finkelstein J, Paz JT, Stehfest K, Fudim R, Ramakrishnan C, Huguenard JR, Hegemann P, Deisseroth K (2011). Neocortical excitation/inhibition balance in information processing
and social dysfunction. Nature.

[bib28] Zhang F, Wang LP, Brauner M, Liewald JF, Kay K, Watzke N, Wood PG, Bamberg E, Nagel G, Gottschalk A, Deisseroth K (2007). Multimodal fast optical interrogation of neural
circuitry. Nature.

[bib29] Zhang F, Prigge M, Beyrière F, Tsunoda SP, Mattis J, Yizhar O, Hegemann P, Deisseroth K (2008). Red-shifted optogenetic excitation: a tool for fast neural control
derived from *Volvox carteri*. Nature Neuroscience.

